# Setting shapes the strain: Untangling alpha-synuclein heterogeneity

**DOI:** 10.4103/NRR.NRR-D-25-01287

**Published:** 2026-01-02

**Authors:** James A. Wiseman, Kreesan Reddy, Glenda Halliday, Birger Victor Dieriks

**Affiliations:** Department of Anatomy and Medical Imaging, University of Auckland, Auckland, New Zealand; Centre for Brain Research, University of Auckland, Auckland, New Zealand; Brain and Mind Centre & Faculty of Medicine and Health School of Medical Sciences, The University of Sydney, Sydney, NSW, Australia

The landscape of α-synucleinopathies is both clinically and pathologically diverse, encompassing Parkinson’s disease (PD), dementia with Lewy bodies (DLB), multiple system atrophy (MSA), and several less common non-classical syndromes, including *PLA2G6*-associated neurodegeneration, *POLG*-associated neurodegeneration, Niemann-Pick type C1, and Krabbe disease. Although each disease is defined by α-synuclein (α-Syn) aggregation, the striking diversity in onset age, anatomical distribution, and clinical course demands an explanation beyond protein misfolding alone. We contend that the key to resolving this paradox is understanding how α-Syn aggregates and their surrounding biological microenvironments co-evolve to shape disease trajectories.

The initiation of α-Syn pathology appears to be influenced by a complex interaction between genetic predispositions and environmental exposures, as suggested by data from genetically predisposed individuals who may be more sensitive to environmental stressors. While conditions like *PRKN*- or *GBA1*-associated PD are genetically driven, environmental factors, including inflammation and toxin exposure, still modulate disease onset and progression. This interplay raises a critical, unanswered question: at what point does α-Syn aggregation transition from an incidental consequence of upstream insults to a self-propagating, strain-driven driver of pathology? Resolving this threshold will be essential for both preventive and disease-modifying interventions (Wiseman et al., 2025b).

A prevailing view is that “chameleonic” nature of α-Syn, including its ability to adopt different structural conformations known as strains, or polymorphs, underpins the diversity of disease phenotypes. Post-translational modifications, lipid interactions, and subcellular compartmentalization each represent potential determinants for modulating strain behavior – determinants that are, in principle, measurable and potentially targetable. Beyond these intrinsic structural properties, the dual role of α-Syn as both a marker and a mediator of disease suggests a dynamic interplay between aggregation and the surrounding microenvironment, spanning cellular, regional, and systemic influences. This environment is not static: the cellular and systemic landscape of a young, pre-symptomatic brain is materially different from that of an aged brain with decades of accumulated stressors, and those differences may bias which strains emerge and dominate.

Consistent with our previous work (Wiseman et al., 2025a, b) and others (Lau et al., 2020), we propose that α-synucleinopathic disease phenotypes are shaped not only by intrinsic α-Syn strain characteristics but also by cell type-specific factors, regional localization, and the broader macroenvironment. As suggested by epidemiological data, we add that the duration of exposure to α-Syn strains is important, including both local and systemic exposures to environmental toxins and other inflammatory mediators (Atterling Brolin et al., 2025). The heterogeneous convergence of these external factors across individuals will likely create a unique pathological milieu that drives the nature of α-Syn aggregation, spread, and pathology. Importantly, we argue for a shift in perspective: from viewing these factors as isolated triggers to embracing a multi-scalar framework that integrates subcellular processes, cell type-specific vulnerability, regional context, and systemic exposures that change over time. This systems-level view emphasizes that α-Syn pathology does not unfold in a uniform substrate but within a complex and evolving biological landscape. Recognizing the role of α-Syn as a dynamic contributor to neurodegeneration within these nested and interacting environments refines our temporal and spatial understanding of disease progression and, crucially, yields explicit, testable predictions for targeted interventions.

**α-Synuclein heterogeneity between diseases: *Lewy body disorders versus multiple system atrophy:*** PD and DLB are often collectively referred to as Lewy body disorders because their hallmark pathological inclusions – Lewy bodies and Lewy neurites – reside in neuronal soma and processes. By contrast, MSA generally lacks these classic Lewy body structures and, instead, features oligodendroglial cytoplasmic inclusions (GCIs) and neuronal cytoplasmic inclusions, within which pathological α-Syn adopts distinct biochemical conformations more aggressive than those typically seen in Lewy body disorders (Peng et al., 2018; Wiseman et al., 2025b). Strain-dependent attributes contribute to these differences. In experimental models, MSA-derived α-Syn strains have demonstrated up to a 1000-fold greater potency in seeding aggregation in oligodendrocytes and neurons compared to PD-derived strains. Whether these seeding efficiencies reflect the *in vivo* processes in humans remains under investigation (Peng et al., 2018). Irrespective of these findings, they support the hypothesis that discrete α-Syn strains, potentially shaped by local cellular milieus (oligodendroglial versus neuronal), may drive more fulminant disease courses such as MSA.

In PD and DLB, α-Syn inclusions remain predominantly cytoplasmic. In MSA, however, α-Syn accumulates not only within the oligodendroglial and neuronal cytoplasm but also within the nuclei (Wiseman et al., 2025a). This dual cytoplasmic-nuclear localization provides further evidence for the role of sub-cellular, cell type-specific, and region-specific processes that potentially alter protein trafficking and degradation pathways, as well as susceptibility to post-translational modification, thereby modulating α-Syn aggregation (Reddy and Dieriks, 2022). While early oligodendroglial dysfunction is observed in MSA, both enteric and nasal vulnerabilities are typical in PD, which likely contribute to the subsequent selective vulnerability of dopaminergic neurons within the substantia nigra pars compacta in the midbrain. DLB, in contrast, is characterized by predominant limbic involvement (especially the hippocampus, amygdala, and cingulate gyrus) rapidly progressing to cortical pathology. Part of this heterogeneity, we contend, arises from different “localized exposures” present at the cellular, regional, or organismal level, for instance, varying pH levels and ionic concentrations within cell types, differing white/grey matter ratios between brain regions, and variable impact of sleep dysfunction.

**Regional heterogeneity of α-synuclein distribution in multiple system atrophy:** Pathological diversity in MSA is well-established, with typically two subtypes – MSA-P (Parkinsonian) and MSA-C (Cerebellar) – characterized by marked subtype-specific loss of non-dopaminergic neurons in the striatonigral (MSA-P) or olivopontocerebellar (MSA-C) systems associated with the pattern of GCIs. As indicated, α-Syn can also deposit within the cytoplasm and nuclei of neurons, illustrating the multicellular and multi-structural complexity of MSA pathology (Reddy and Dieriks, 2022).

***Neuronal and oligodendroglial pathology:*** Although GCIs are pathognomonic for MSA and concentrate in regions of neuronal degeneration, the widespread presence of neuronal α-Syn inclusions suggests that disease progression may depend, at least in part, on abnormal α-Syn trafficking between neurons and oligodendroglia (Reddy and Dieriks, 2022; Wiseman et al., 2025a). Which cell type drives neurodegeneration, however, remains a key unknown; on the one hand, the formation of nuclear α-Syn filaments in neurons rapidly causes their demise (Reddy and Dieriks, 2022; Wiseman et al., 2025a), while GCIs cause primary oligodendroglial dysfunction by altering myelin and protein homeostasis but not killing the cell. Notably, variation in the strains of MSA fibrils is well documented (Schweighauser et al., 2020), with protein interactions and post-translational modifications causing additional variations (Kametani et al., 2024). There is evidence that these different α-Syn strains in oligodendrocytes in MSA are abnormally trafficked, resulting in a synergistic assault of oligodendroglial pathology and neuronal cell loss, resulting in the accelerated decline observed in MSA patients (reviewed in Reddy and Dieriks, 2022).

***Future direction:*** To determine how these localized exposures shape strain evolution, *in vitro* and *in vivo* models should expose neuronal and oligodendroglial cultures to controlled microenvironmental variables (pH, ionic composition, lipid content), while modeling both nuclear and cytoplasmic α-Syn aggregation. Structural and seeding assays of the resulting fibrils, followed by comparison to patient-derived strains, could validate which environmental and subcellular parameters most strongly influence strain conformation.

**Regional heterogeneity of α-synuclein distribution in Parkinson’s disease:** PD has historically been described using Braak staging, where α-Syn pathogenesis always initiates in either the dorsal motor nucleus of the vagus nerve and/or the anterior olfactory nucleus, and subsequently spreads in a predictable pattern throughout the brain over decades. This vagal and olfactory pathology has since been proposed to initiate peripherally in a “body-first” pattern from tissues directly exposed to pathogenic insult (e.g., gastrointestinal or olfactory epithelium) before subsequently ascending the neuroaxis. Recent findings do not agree with this simple one-model-fits-all theory and propose alternate patterns of neuronal spreading. Some patients show a brainstem initiation of α-Syn accumulation with a slower spread, while other patients show a more central “brain-first” pattern, in which pathology originates within the central nervous system itself (reviewed in Wiseman et al., 2025b). These differing sites of seeding origin, combined with environmental exposures, suggest that local microenvironments play a critical role in shaping the specific strain of α-Syn that forms and in directing the trajectory of disease progression (**Figure 1**).

**Figure 1:**
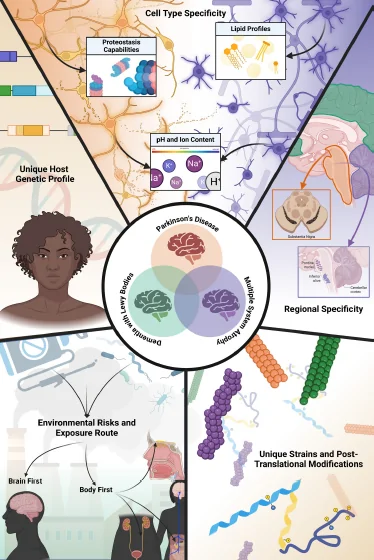
Schematic overview of the multifactorial determinants shaping α-synuclein (α-Syn) strain diversity. α-Syn can adopt various structural conformations (strains or polymorphs), contributing to the phenotypic heterogeneity observed across α-synucleinopathies. This structural plasticity is modulated by post-translational modifications (e.g., phosphorylation, truncation), which expand both the structural and functional diversity of misfolded species. Beyond these intrinsic properties, extrinsic factors – including cell type-specific mediators, regional brain environments, and systemic exposures (e.g., environmental toxins, inflammation, and peripheral entry routes) – play critical roles in shaping α-Syn aggregation, spread, and toxicity. The interplay between α-Syn’s intrinsic features and the evolving microenvironment underlies the clinical and pathological diversity within and across synucleinopathies. Created with BioRender.com.

These multifaceted trajectories of α-Syn pathogenesis through the brain underscore the heterogeneity within PD itself. Genetic predispositions (e.g., *PINK1*, *LRRK2*, and *SNCA* mutations) can modulate how α-Syn is handled within neurons. Simultaneously, environmental triggers, including pesticides, microbial metabolites, and viral exposures, influence local inflammatory responses or protein misfolding cascades that may precede or enhance α-Syn aggregation (**Figure 1**). The point at which α-Syn aggregation transitions from an incidental by-product to a self-propagating driver will further shape disease trajectory. The interplay of these factors likely impacts onset, regional spread, and progression rates even among individuals with the same PD diagnosis, underscoring the need for refined classification systems incorporating genetic, environmental, and molecular markers of α-Syn pathology. This supports redefining PD not as a single entity but as a syndrome of distinct subtypes, potentially delineated by patterns of α-Syn propagation, genetic risk, or exposure history.

***Future direction:*** To resolve whether “body-first” and “brain-first” PD represent distinct strain–environment combinations, longitudinal sampling from prodromal individuals (e.g., REM sleep behavior disorder patients) should combine seed amplification assays from multiple tissues and multiple brain regions with deep genetic and environmental profiling. This would test whether strain structure at disease onset predicts subsequent anatomical spread.

**Regional differences using seed amplification assay approaches:** A growing body of work employs seed amplification assays, such as real-time quaking-induced conversion, to differentiate α-Syn polymorphs extracted from postmortem brains or patient samples (e.g., cerebrospinal fluid or skin). These studies reveal not only disease-specific seeding profiles distinguishing PD from MSA-derived α-Syn, but also striking regional variation in aggregation kinetics within individual brains (Martinez-Valbuena et al., 2022; Wiseman et al., 2025c). Notably, PD-derived α-Syn fibrils often exhibit greater structural heterogeneity between brain regions than those derived from MSA (Strohäker et al., 2019; Wiseman et al., 2025c).

One overlooked aspect is varying lipid compositions across brain regions. Not only is fibrillization of α-Syn strongly enhanced in the presence of lipid vesicles, but the three-dimensional structure of α-Syn filaments also changes depending on the binding of lipid molecules with fibrils (Frieg et al., 2022). As a result, differential region-specific interactions of α-Syn filaments with both lipids and proteins may play a core role in driving the regional heterogeneity of α-Syn pathology and, therefore, the diversity of clinical phenotypes observed in PD, such as differences in motor phenotype or cognitive trajectory. Whilst these seeding assays do suggest conformational diversity in PD, it is important to note that such methodologies may not fully capture the complexity of *in vivo* strains due to potential methodological artefacts (Strohäker et al., 2019). While experimental models help isolate strain-specific effects by minimizing external variables, it is ultimately the complex interplay between strain properties, local environment (such as interacting proteins, lipids, and genetic backgrounds), external factors (such as systemic and macroenvironmental influences), as well as temporal variations throughout disease progression, that likely drives disease heterogeneity (**Figure 1**). As such, this hypothesis requires further validation in longitudinal clinical and neuropathological studies to identify potentially pivotal changes.

These intra-individual regional differences may also reflect microenvironmental variables such as proteostatic capacity or selective neuronal vulnerability. In PD, pathology diverges markedly between the substantia nigra pars compacta and hippocampus. The substantia nigra pars compacta, with its high oxidative stress and dense catecholaminergic population, may promote one α-Syn polymorph, whereas the hippocampus, where N-terminal α-Syn inclusions are prominent in astrocytes, may favor another. Similarly, in MSA, regional heterogeneity likely results from differences in myelin, oligodendroglial homeostasis, and combined glial and neuronal involvement. The cerebellum exhibits predominantly GCIs, while the medulla, particularly the inferior olivary nucleus, displays a more mixed pathology with substantial neuronal involvement (Wiseman et al., 2025a).

***Future direction:*** Multi-region sampling within the same brain, paired with lipidomic and proteomic profiling, could establish causal links between local biochemical milieu and α-Syn fibril conformation. Importantly, assays should be run in parallel in multiple centers to ensure reproducibility and eliminate methodological artefacts.

**Role of environmental influences in driving α-synuclein heterogeneity:** The ever-changing exogenous factors that dynamically shape the microenvironment in which α-Syn misfolding initiates and progresses are likely core contributors to α-Syn heterogeneity. Evidence suggests that these factors include pesticide exposure and pollution, microbiome interactions, and varying inflammatory states. Such factors may also contribute to the selective vulnerability of specific cell types and influence the efficiency of aggregate propagation between cells and brain regions.

***Exposure pathways:*** The involvement of the olfactory and enteric systems in PD and DLB, and the urinary system in MSA, underscores how external insults (pesticides, pollutants, and pathogens) may infiltrate the nervous system directly via nasal, gut, or urinary routes. These external insults can spark initial α-Syn misfolding events in peripheral neurons, which subsequently spread to the CNS.

***Microenvironmental milieu:*** Once inside the CNS, α-Syn strains confront different local conditions with varying ion content and acidity. For instance, oligodendrocytes in MSA may exhibit distinct lipid profiles, proteostatic capacities, pH, and inflammatory states compared to neurons in PD or DLB, fostering alternative aggregation routes (Reddy and Dieriks, 2022). Our recent work on the subcellular localization of α-Syn pathology in neurons and oligodendrocytes directly supports the notion that the microenvironment, not just at the cellular but also at the subcellular level, critically shapes the aggregation landscape. For instance, the nuclear presence of α-Syn in MSA neurons raises questions about nuclear trafficking and the compartment-specific mechanisms that may influence strain evolution or toxicity (Wiseman et al., 2025a).

***Post-translational modifications:*** Genetic and environmental stressors can alter redox balance, kinases, and proteases, influencing phosphorylation or truncation patterns of α-Syn. These modifications, in turn, can yield distinct α-Syn fibril conformations with unique toxic and seeding profiles (Yoo et al., 2022).

Together, these observations support a model in which α-Syn heterogeneity arises from the interplay between strain diversity, region- and cell type-specific vulnerability, host genetics, environmental exposures, and their evolution over time.

***Future direction:*** Controlled exposure studies in α-Syn transgenic animal models, in which single environmental variables (e.g., defined pesticide, microbiome composition, and inflammatory cytokine profile) are manipulated, could reveal whether specific stressors reproducibly bias strain selection. Structural and functional assays of resulting fibrils would establish mechanistic links between environment and conformation.

**Conclusion:** The heterogeneity of α-synucleinopathies is not simply a reflection of distinct diseases but a consequence of how α-Syn interacts with its evolving biological context, from subcellular compartments to systemic environments. Strains do not emerge in a vacuum; they are sculpted by the conditions in which they form (**Figure 1**). However, once formed, they also feed back into their environment, altering cellular function, inflammatory tone, and network vulnerability in strain-specific ways. This conceptual shift – from viewing α-Syn as a static pathological entity to a dynamic participant in a reciprocal disease process – reframes both prevention and treatment. From this perspective, we identify three immediate priorities for the field: (1) define the “conversion threshold” at which aggregation becomes self-propagating; (2) map context–strain interactions across different dimensions of organisation (subcellular, cellular, and regional) using harmonized, reproducible assays; and (3) design context-matched interventions that target environments favoring pathogenic strain emergence, including modifiable systemic factors. If strain evolution is contingent on physiological and environmental conditions that can be measured and modified, intervention could begin long before the first fibril appears.

*KR is funded through an Auckland Medical Research Foundation Doctoral Scholarship. BVD is funded by a Health Research Council of New Zealand Hercus Fellowship (21/034), the School of Medical Science, University of* *Auckland, and Te Tītoki Mataora (3729090 & 3729858). GH is funded by the National Health and Medical Research Council of Australia (Investigator Grant 1176607). The funders had no role in study design, data collection and analysis, publication decisions, or manuscript preparation.*

## References

[R1] Brolin KA, Schaeffer E, Kuri A, Rumrich IK, Schumacher Schuh AF, Darweesh SK, Kaasinen V, Tolppanen A, Chahine LM, Noyce AJ (2025). Environmental risk factors for Parkinson’s disease: a critical review and policy implications. Mov Disord.

[R2] Frieg B, Antonschmidt L, Dienemann C, Geraets JA, Najbauer EE, Matthes D, de Groot BL, Andreas LB, Becker S, Griesinger C, Schröder GF (2022). The 3D structure of lipidic fibrils of α-synuclein. Nat Commun.

[R3] Kametani F, Tahira M, Takao M, Matsubara T, Hasegawa K, Yoshida M, Saito Y, Murayama S, Hasegawa M (2024). Analysis and comparison of post-translational modifications of α-synuclein filaments in multiple system atrophy and dementia with Lewy bodies. Sci Rep.

[R4] Lau A, So RWL, Lau HHC, Sang JC, Ruiz-Riquelme A, Fleck SC, Stuart E, Menon S, Visanji NP, Meisl G, Faidi R, Marano MM, Schmitt-Ulms C, Wang Z, Fraser PE, Tandon A, Hyman BT, Wille H, Ingelsson M, Klenerman D, Watts JC (2020). α-Synuclein strains target distinct brain regions and cell types. Nat Neurosci.

[R5] Martinez-Valbuena I, Visanji NP, Kim A, Lau HHC, So RWL, Alshimemeri S, Gao A, Seidman MA, Luquin MR, Watts JC, Lang AE, Kovacs GG (2022). Alpha-synuclein seeding shows a wide heterogeneity in multiple system atrophy. Transl Neurodegener.

[R6] Peng C, Gathagan RJ, Covell DJ, Medellin C, Stieber A, Robinson JL, Zhang B, Pitkin RM, Olufemi MF, Luk KC, Trojanowski JQ, Lee VM-Y (2018). Cellular milieu imparts distinct pathological α-synuclein strains in α-synucleinopathies. Nature.

[R7] Reddy K, Dieriks BV (2022). Multiple system atrophy: α-Synuclein strains at the neuron-oligodendrocyte crossroad. Mol Neurodegener.

[R8] Schweighauser M, Shi Y, Tarutani A, Kametani F, Murzin AG, Ghetti B, Matsubara T, Tomita T, Ando T, Hasegawa K, Murayama S, Yoshida M, Hasegawa M, Scheres SHW, Goedert M (2020). Structures of α-synuclein filaments from multiple system atrophy. Nature.

[R9] Strohäker T, Jung BC, Liou SH, Fernandez CO, Riedel D, Becker S, Halliday GM, Bennati M, Kim WS, Lee SJ, Zweckstetter M (2019). Structural heterogeneity of α-synuclein fibrils amplified from patient brain extracts. Nat Commun.

[R10] Wiseman JA, Halliday GM, Dieriks BV (2025). Neuronal α-synuclein toxicity is the key driver of neurodegeneration in multiple system atrophy. Brain.

[R11] Wiseman JA, Reddy K, Dieriks BV (2025). From onset to advancement: the temporal spectrum of α-synuclein in synucleinopathies. Ageing Res Rev.

[R12] Wiseman JA, Turner CP, Faull RLM, Halliday GM, Dieriks BV (2025). Refining α-synuclein seed amplification assays to distinguish Parkinson’s disease from multiple system atrophy. Transl Neurodegener.

[R13] Yoo H, Lee J, Kim B, Moon H, Jeong H, Lee K, Song WJ, Hur JK, Oh Y (2022). Role of post-translational modifications on the alpha-synuclein aggregation-related pathogenesis of Parkinson’s disease. BMB Rep.

